# Hospital and Institutional News

**Published:** 1918-08-31

**Authors:** 


### HOSPITAL AND INSTITUTIONAL NEWS.

### AN INSTRUCTION MR. CHURCHILL SHOULD ISSUE.

In attempting to organise weekly contributions from workpeople, hospitals at the present time are confronted with a serious difficulty. The munition works now occupy a principal place in the industrial life of the nation, and they inevitably supply a large number of accident and other cases to the hospitals. Meanwhile the cost of hospital maintenance continues to rise, and general efforts are being made to organise sectional subscriptions from workpeople and their employers. Since this support from private firms was a recognised source of income before the war, efforts have been made to establish it on a firm basis, and this effort has been welcomed on the whole alike by the workpeople and their employers. To complete the scheme a like organisation is required from munition works, but this cannot be secured without the co-operation of the Minister of Munitions. The officers in charge of the Government works often refuse to sanction or support a weekly contribution from their workpeople, though they do not hesitate to send a regular stream of patients to the hospitals' doors. Unless the support of Mr. Churchill is secured, this injustice will not be remedied. Nor does the difficulty concern Government works alone. Our readers will recall the request of certain firms to Mr. Lupton, the chairman of the General Infirmary of Leeds, to use his influence to permit their subscriptions to hospitals to be charged against their working expenses before the question of excess profits was discussed. As The Hospital recorded on July 20, a similar arrangement is already in force in regard to clubs for munition workers, but after many delays Mr. Lupton was informed that the suggestion could not be conceded without a new Act of Parliament. In *that* case, the yield to the Exchequer being involved, there might be reasonable excuse for delay or hesitation, but no such excuse can be offered for the Minister of Munitions. Mr. Churchill has nothing to prevent him from issuing an instruction to the officers in charge of the munition works to encourage the workmen to subscribe a weekly penny from their wages to the hospitals on which they and their families depend in the moment of accident and the crisis of disease.

### MORE BEDS FOR CIVILIANS AT BOSCOMBE.

A new ward has been opened at the Boscombe branch of the Royal Victoria and West Hants Hospital, Bournemouth. It is for the treatment of sick and wounded soldiers, and accommodates thirty-two beds. The Mayor of Bournemouth, in opening the new ward, called the "Bournemouth Ward," said that he was glad that this new ward would release one of the permanent wards inside the hospital for the treatment of civilian patients. The chairman of the house committee, Mr. E. H. Bellairs, commented upon the fact that the new erection was the outcome of their experience in other wards, and they could claim, therefore, that it was the latest in the process of evolution. The Bournemouth War Relief Committee has contributed £500 to the cost of the new building, and the hospital has supplied the remainder. Visitors and inspectors have admired the efficiency attained at this hospital.

### AUTOGRAPHS AND CHARITY.

One hundred guineas were realised last week at Barmouth in aid of the Welsh hospitals, London, by the sale of a five-pound note bearing the autograph of the Prime Minister. The idea is to maintain Barmouth beds in these institutions, and the sum immediately aimed at for this purpose was more than secured. Everything is now sold for the benefit of charities, but in England autographs have been the province of the genuine collector, since the bombardment of a favourite actor or actress has been made by private admirers and not by those making an impartial collection. In America, however, every autograph has its price, and before the war it used to be an amusement of American writers and journalists to trot round to the nearest autograph shop and measure their reputation by the rise or fall of the price of their signatures.

### HOSPITAL WORK IN PALESTINE.

Since the British occupation of Southern Palestine, at Jerusalem, Bethlehem, Jaffa, Gaza, and Hebron hospitals and dispensaries have been established anu health bureaux set up, where records are kept of births, deaths, and infectious diseases, and where advice is given to the populace. Everywhere the streets are swept and kept clean, and a regular supply of pure water has been provided for many towns. These measures have brought about a marked improvement in the public health of Southern Palestine as compared with its condition a year ago. Epidemics have been checked, and the only infectious disease that is at all prevalent now is malaria. A few isolated cases of typhus remain, but though an outbreak of small-pox was feared because of the discontinuance of vaccination, only a handful of cases has occurred. Cholera also has been dealt with. The death-rate even in Jaffa and Jerusalem is now understood to be lower than in most European cities, a fine comment on the British occupation of Palestine.

### UNWILLING VOLUNTEERS.

In a lecture on "The Advances of Bacteriology" at King's College, Dr. Browning, director of the Bland-Sutton Institute at the Middlesex Hospital, said that the treatment of trench-fever, now such a serious problem, could be studied only by the inoculation of human beings. The investigations of a special committee which was engaged upon the subject at the Hampstead Military Hospital were hindered by a lack of people willing to undergo inoculation. Volunteers apparently are wanted and are to seek, but though individuals can be found for most things, it cannot be denied that the rank and file are suspicious of inoculation in any form, and this fact no doubt explains the unwillingness referred to. The heroic self-sacrifice of eight British soldiers in submitting themselves to experiment under the care of Sir David Bruce's Committee on Trench Fever, which we described in our issue of June 29, page 282, should stimulate civilians and others to come forward.

### A FINE EXAMPLE OF CONTINUITY.

THE Aldershot Hospital has achieved a record by celebrating its twenty-first anniversary with so many officials still in its service who were associated with it at its opening on August 13, 1897. Among these are the President, Mr. W. T. Robertson, J.P., C.C.; two hon. members of the medical staff, Dr. F. Stroyan and Dr. J. H. Gibson; the Hon. Treasurer, Mr. A. J. Andrews; the Hon. Solicitor, Sir William Foster; the Hon. Auditor, Mr. R. Simmonds, J.P.; the Hon. Secretary, Mr. W. Wren, and the matron, Miss Gregory. In addition to a handsome testimonial to the matron, the committee have presented to Mr. Wren a gold watch with suitable inscription. The hospital has rendered excellent service to the town during its twenty-one years of existence, and in particular since war began has treated a large number of soldiers' wives and children.

### A NEW COTTAGE HOSPITAL.

THE scheme for a cottage hospital for the borough of Flint is likely to be realised. A building, valued at £3,000, has been offered as a gift to the hospital committee by Messrs. Courtaulds on condition that an endowment fund of at least £3,000 should be raised in the neighbourhood. Such a fund would give the nucleus which further gifts might extend. Already over £2,000 has been subscribed, and, thanks to the active support of Dr. J. H. Williams, the medical officer of health to the borough, the remainder is likely to be forthcoming. The chairman of the hospital committee is Mr. J. R. Alexander, who is a vigorous supporter of the scheme.

### A WOMAN HOSPITAL MEDICAL SUPERINTENDENT.

THE Women's Suffrage Conference at Johannesburg on Friday, July 5, passed an address of congratulation to Dr. Mary Gordon on her acting appointment as senior medical officer of the Johannesburg General Hospital. It was pointed out to the Suffrage Conference that this was the first appointment of a medical woman made by the Johannesburg Hospital Board, and an additional address of congratulation was forwarded to the board on the appointment of Dr. Mary Gordon.

### CRITICISM OF PROVISION FOR TREATMENT OF TUBERCULOSIS.

ADMINISTRATIVELY the old familiar features of the anti-tuberculosis campaign in this country continue to manifest themselves, with, of course, the war-time addition of the problem of tuberculous combatants. The disease has become more prevalent, in face of all the measures based on the overweening importance of infection; there is consequent disappointment, expressed, for instance, at the opening of the New Central Institute for Tuberculosis at Belfast. No good will come of blinking this fact. The increase is much too large and sudden to be attributable to increased refinement of diagnosis. What is incumbent upon all concerned is to recognise that impairment of the general health is a tuberculosis-producing factor of the vastest potency, hitherto much underrated, and to modify anti-tuberculosis methods accordingly. With the increase in tuberculosis comes the call for more sanatorium beds, heard, for instance, from Staffordshire. Elsewhere there are complaints of existing institutions, complaints made by patients and made much of by lay investigators. So long as the call just mentioned is responded to by fitting out numerous small houses, small-pox huts, etc., as so called sanatoria, instead of centralising in large buildings, if necessary enlarging existing ones, and so long as resident sanatorium superintendents are mere house physicians, so long will British public sanatoria remain in their present backward state, and undisciplined patients draw the long bow. We note that a tuberculosis officer in one district sought to become president of the local recruiting board, to devote five mornings and five afternoons weekly to his new duties, to be paid extra emolument at the rate of over £500 a year for them, and presumably to accommodate his regular work to the new calls upon his time. The full committee very rightly refused such a proposal. We express no opinion in this particular instance, but the elective vagaries of local municipal bodies have often enough put in charge of dispensaries men with minimal experience of tuberculosis work, and no keenness at all for it. As to the sad case of tuberculous combatants, it is sensibly enough becoming recognised that they must be more unpromising patients than civilians. If only from pity, or rather justice, the same standards of selection for admission to the sanatorium cannot be applied. Accordingly, the results of treatment must be judged separately, especially as soldiers leave sanatoria very readily in winter, and where male medical officers are absent on military duty.

### REFRESHMENTS FOR OUT-PATIENTS.

THE payment of the travelling expenses of out-patients and the provision of light refreshments for them are suggested by the City of London Guardians, who have placed at the disposal of the Metropolitan Asylums Board a small infirmary at Thavies Inn for the treatment of women, as part of the campaign for dealing with venereal disease. An outdoor clinic has been established for women who have been discharged from the infirmary, but who still need treatment and surveillance as they come from all parts of London. The proposal has been submitted to the Local Government Board.

### PHYSICAL CLINICS ON THE INCREASE.

SOME years ago every hospital was struggling to secure for itself an $x$-ray department; now, when treatment by means of drugs has yielded so much to treatment by massage, electricity, and exercises, a frequent development in voluntary hospitals takes the shape of a fully equipped exercise department.

The next extension in this respect will possibly be at the Sunderland Royal Infirmary, where Mr. Hiram Craven, a generous local supporter, has given £500 as the first donation of a fund to provide a complete physical clinic, in which the various forms of baths, the different kinds of manipulations, both voluntary and by means of mechanical apparatus, will be provided. The physical clinic will be for the benefit of both military and civilian patients who are in need of the different forms of treatment.

### NEW ZEALAND HOSPITAL FINANCE.

Hospital expenditure in this country has increased enormously during the war, and from reports that reach us from time to time the hospitals of the Dominions find themselves very much in the same financial position. There is this difference, however, that while here the increased burden falls upon the comparatively faithful few, in Australia, New Zealand, etc., it is a question of larger rates and taxes, and the facts obtain larger publicity. In New Zealand it will be remembered that the money required to run the hospitals comes from the State, from municipalities, and thirdly (in a very small degree) from the charitable public. The municipality, as a rule, kicks when it is asked to give more. Certainly it has a real grievance, as it has no voice in the election of the hospital board who spends the money. In Wellington, for the upkeep of the hospital, the levy was £23,055, or 6s. 3½d. per head, for 1918, as compared with £16,572, or 4s. 6¼d. per head, for 1917, and the city is asked to pay 72 per cent. of the total revenue required by the hospital board.

### SUGGESTIONS FOR RETRENCHMENT.

The City Council accordingly are disposed to be rather critical as regards the general system of raising and spending hospital funds, and ask why Wellington Hospital should be so much more expensive to manage than Auckland Hospital. The total charges in the first-named institution, according to latest returns, were £115, while at the second the figure was £95. A champion of the Wellington Hospital Board, in an account of an interview published in a local paper, is reported to have claimed that it is not fair to judge a hospital by the expenditure of a single year, and that, taking the last five years, the figures are for total maintenance per occupied bed: Auckland £97, Wellington £103, which is by no means a very acute difference. Capital charges not entering into the calculation, we are not able quite to follow the gentleman's reasoning; indeed, from the figures it appears to be chiefly under the heading of salaries and wages that the two hospitals differ so widely, the cost per occupied bed in Auckland being £28, against £54 in Wellington. It is quite easy to get together an unnecessarily large and expensive staff, and it is in this department of the Wellington Hospital finances that a reformer would probably first seek for suggestions for retrenchment.

### TO PAINT OR NOT TO PAINT.

Soon after the outbreak of war many public authorities decided to defer the painting and thorough cleansing of wards in their hospitals, in the belief that at the outside two years' delay would make no material difference. Unfortunately, the war has already continued twice as long as was anticipated, but the necessary painting has been deferred until in many cases it has now become absolutely essential for the preservation of the buildings that the work should be done this year. The Wandsworth Board of Guardians, for instance, was informed by their medical superintendent, Dr. W. L. MacCormac, that in his opinion it was essential that the lying-in ward should be painted, but the Works Committee came to the conclusion that it was unnecessary, a view which was supported by the Guardians. When a request of this character is made by a medical superintendent, it may be taken that it is made in good faith and with a wish to promote the best interests of everybody concerned. Such being the case, it is surprising that the Wandsworth Board of Guardians have not fallen in with the request of Dr. MacCormac.

### THE RETENTION OF A SUFFICIENT STAFF.

Public authorities have as a general rule only appealed for the exemption from military service of employees who are essential. Occasionally they have decided not to ask for any officer of military age to remain, and the Bermondsey Guardians have adopted this policy. For some time they supported the claim for exemption for the steward of their infirmary, but, having come to the conclusion that no man was irreplaceable, they instructed their clerk not to appear before the Tribunal when the claim was heard. It was evident from the observation of the National Service representative (Lieut. J. Albert Davis), who had visited the infirmary and satisfied himself that the steward was doing essential work, that the man was indispensable, especially as he came within the scope of the certified occupation list. The Tribunal agreed, and granted the steward conditional exemption so long as he remained in the same occupation. It is evident that the Guardians did not appreciate that as the steward was in the certified occupation list he could obtain a similar situation with another Poor-Law authority, and secure the same exemption on his own initiative without the support of his employers.

### THE DOWNS SANATORIUM.

Dr. James Wall, who has completed his probationary period in the Sanatorium Service of the Metropolitan Asylums Board, has been confirmed in his appointment as medical superintendent of the Downs Sanatorium.

### A LUCKY SURGICAL INSTRUMENT MAKER.

In spite of an energetic attempt to stop the drawing for prizes in a lottery for the benefit of war charities at Nottingham, when the Watch Committee refused the appeal of the Methodist Brotherhood to intervene, the prize list was duly announced. Sixty thousand tickets were said to have been sold, and the winner of the first prize, a £150 War Bond, was an assistant in a surgical instrument maker's shop at Nottingham.

### A COLLECTIVE FEE FOR A BATCH OF SCHOOL CASES?

In consequence of a resolution of the Kent Hospital Committee with regard to fees to be paid to the medical staffs of hospitals, the Kent County Council has received from the Kent Ophthalmic Hospital, and from some of the other hospitals in the county, notice to terminate the existing arrangements for the treatment of cases recommended by the school medical inspectors. The Education Committee is anxious not to suspend any of the work of medical treatment, but feels that the new scale of charges now proposed is more than it can accept. It thinks that if surgical cases could be presented in batches of half-a-dozen it would not be unfair to ask the operator and anæsthetist to accept a lower fee than those now proposed. The Education Committee has invited the hospital committee to appoint two or three representatives to meet the representatives of the Education Committee to discuss the matter.

### COMMANDEERING HOSPITALS FOR INFECTIOUS DISEASE.

The Health Committee of the Greenwich Borough Council has considered a letter from the Metropolitan Asylums Board stating that recently, under pressure from the Army Council, it has agreed to hand over two large hospitals containing over 1,500 beds to the military authorities. These hospitals are in addition to four of the Board's hospitals already commandeered. With a view to making the best possible use of the beds in the remaining hospitals local authorities are asked to assist by selecting for admission only those patients who urgently require hospital treatment, or those whose circumstances imperatively call for their removal. The Board also suggests that mild cases of scarlet fever and of bacteriological diphtheria might be treated at home, of which course the Local Government Board approves. The Greenwich Health Committee views with some apprehension a condition of things which may arise owing to so serious a curtailment of hospital accommodation for cases of infectious fever. It is informed that it is the slight cases which are often most infectious. While the committee would hesitate to oppose accommodation for wounded soldiers in these hospitals, it thinks that under the circumstances the Council should enter a protest against the beds available for infectious cases being so seriously reduced in number.

### SALARIES AND COMPETITION.

Evidence accumulates that it is the rate of wages attached to Government posts which makes it difficult for institutional authorities to retain their old staffs or to engage new officers. The Metropolitan Asylums Board has had considerable difficulty, and is said to have lost an attendant because the man was able to secure for new work at the Arsenal double the wages that he received from the Board. Already the scale of salaries has been raised in consequence, but we hope that the moral will not be lost. Hospital salaries have had a long way to make up, and though Government posts will not last for ever, they have given a useful incentive to institutional managers to improve the salaries of the staffs all round.

### A REMARKABLE BODY OF NURSES.

As soon as sufficient funds shall have been collected, a home for totally disabled Roman Catholic sailors and soldiers will be opened at Ealing. The house which has been secured, with considerable grounds, has been officially inspected and publicly approved by Sir Arthur Griffith Boscawen, M.P. Half the sum total required, which amounts to £25,000, is needed for immediate alterations and equipments. The most interesting fact about the institution, which will be known as St. David's Home, is the arrangements which are being made for the nursing of these wholly dependent patients. The promoters believe that a special vocation is required for the nursing of those whose every need must be ministered to by others, and the patients will be nursed by the Franciscan Missionaries of Mary, an Order whose rule enjoins them continually "to be busied with the miseries of life." Some of the nurses, it is stated, have been engaged in France, where they have had direct experience of war and its horrors. The chairman of the committee is Lady Anne Kerr, O.B.E., the secretary Mrs. Wallis, and the treasurer Mrs. Passmore, who will be glad to receive donations at 3E Hyde Park Mansions, N.W. 1. That the nursing of the sick is first a vocation and secondly only a profession we need to be reminded, especially in these days, and the present army of nurses should be reminded of its high calling by the existence and work of this Order, which in peace draws its patients largely from among the lepers and the plague-stricken, and in war is now claiming the most stricken class of sufferers as its own peculiar care.

### THIS WEEK'S DRUG MARKET.

The upward movement in the price of menthol is one of the main features of the market, but the rate of the advance has a tendency to slow down. It is rather curious that the price of menthol, which until the last week or so had only been affected by the war to a slight extent, should have begun to advance at a time when the majority of drugs have temporarily ceased their violent fluctuations. Japanese dementholised peppermint oil is also dearer, and the same applies to star aniseed oil. The high price of camphor is firmly maintained, with the prospect of a further advance in the near future. A small business continues to be done in quinine at the advanced rate. There is no change in bromides. The price of aspirin has an upward tendency, and the same applies to salicylic acid and sodium salicylate. Available supplies of benzoic acid and sodium benzoate are inadequate to meet the demand, and high prices are asked. Supplies of sugar of milk are expected to arrive shortly, and prices may be lower in consequence.

